# The Impact on Dental Staining Caused by Beverages in Combination with Chlorhexidine Digluconate

**DOI:** 10.1055/s-0041-1742123

**Published:** 2022-02-23

**Authors:** Sandra Sarembe, Andreas Kiesow, Jonathan Pratten, Corinne Webster

**Affiliations:** 1 Fraunhofer Institute for Microstructure of Materials and Systems IMWS, Halle, Germany; 2 GSK Consumer Healthcare, Weybridge, United Kingdom

**Keywords:** chlorhexidine, dental staining, beverages, color measurements, SEM

## Abstract

**Objectives** There are several hypotheses regarding how chlorhexidine (CHX) digluconate causes staining with the role of beverages, specifically the precipitation of anionic dietary chromogens onto adsorbed cations, the most probable cause. The aim of this study was to investigate and compare the staining potential of common beverages using an *in vitro* staining and brushing model to better understand the interactions between chromogens from different beverage categories and the teeth.

**Materials and Methods** Human enamel samples were exposed to a cyclic treatment of artificial saliva and 0.2% CHX mouthwash combined with a range of beverages, with and without brushing, simulating a period equivalent to 2 weeks. Eleven beverages were tested: diet coke, diet lemonade, white wine, red wine, lager beer, black tea, coffee, black tea with milk, coffee with milk, ginger and lemon infusion, and water. Toothbrushing was performed in a brushing simulator with toothpaste and also with water. Colorimetric differences were determined by ΔE using a VITA Easyshade dental spectrophotometer. Statistical analyses were performed by one-way analysis of variance with post hoc Tukey's honestly significant difference test and Levene's test.

**Results** Black tea and red wine produced highest staining, which agrees with the literature. Significant staining was also observed for a ginger and lemon infusion, coffee, coffee with milk, tea with milk, and lager beer compared with water ( *p* < 0.05). The staining potential of diet coke in combination with brushing appeared to be connected to its low pH. Both white wine and diet lemonade produced stain comparable to the water control. After treatment with high staining beverages, scanning electron microscope evaluation confirmed the formation of a surface layer. The mechanical resistance of the stain differed depending on the beverage, black tea stain was the most resistant. The addition of milk to tea and coffee considerably modified the stain layer and the adhesion to the tooth surface.

**Conclusion** The data may help demonstrate that appropriate user guidance can avoid stain and in turn help improve user compliance during short-term use of this gold standard antimicrobial treatment.

## Introduction

For more than 40 years, 0.2% chlorhexidine (CHX)-containing mouthwashes have consistently been shown to inhibit the formation of plaque to give a significant clinical improvement in the development of gingivitis 1 and furthermore recognized as the gold standard in preventing infection. 2 However, reported side effects may affect patient compliance. The most common side effect of CHX digluconate in oral use is tooth and tongue staining, which appears to be an unavoidable consequence of clinical efficacy. 3 4 It has been suggested that this patient-to-patient variability is linked to external factors such as patient's diet. 5

Numerous studies with model systems have shown that dietary chromogens are at least partly responsible for stain. Many of these materials including tea, coffee, and tobacco are known to cause staining even in the absence of CHX. 6 As indicated by most of the studies, the most probable cause of staining is the precipitation of anionic dietary chromogens onto adsorbed cations. 6 7 8 This means that polyphenols, which are anionic and can be found in dietary substances, can react with cations adsorbed to surfaces, including cationic antiseptics such as CHX to then form a stain. 6 8

Avoiding strongly coloring beverages is one way of reducing the risk of stain while using CHX-containing products. Therefore, the aim of this study was to investigate the staining propensity of representative beverages in combination with CHX in an *in vitro* test model to obtain a data basis which is useful to produce a simple recommendation for alternative beverages to avoid staining during a CHX treatment. Attention was paid that the beverages covered a wide range of the relevant properties regarding pH value and content of chromogens. From the different, selected beverage types available on the European market, only one representative product per category was chosen. The *in vitro* test model was developed to simulate an application period of 2 weeks including a cyclic test procedure mimicking daily use. In addition to the pure exposure treatment, a brushing step was integrated to assess the impact of mechanical interaction by brushing.

## Materials and Methods

### Sample Preparation

Extracted human molars were used for preparing appropriate specimens. After the removal of residual tissue, the teeth were stored for 24 hours in 10% hydrogen peroxide (Sigma-Aldrich, Germany). The roots were separated from the crowns by a water-cooled diamond saw (Minitom, Struers, Germany). The crowns were sectioned in halves. In total, 132 samples (halved tooth crowns) were etched in 2% citric acid (Sigma-Aldrich, Germany) for 1 minute and fixed with a two-component epoxy resin adhesive (UHU plus endfest 300, UHU, Germany) to a slide.

### Artificial Saliva

Artificial saliva was prepared as previously described. 9 Prior to every use, it was stirred to homogenize the precipitated contents. The artificial saliva was renewed after every seventh cycle.

### Chlorhexidine Treatment

Chlorhexamed Forte, alcohol-free (GlaxoSmithKline Consumer Healthcare GmbH & Co. KG, UK) was selected as the 0.2% w/v CHX treatment. It was used as provided: 10 mL for 1 minute twice daily for a duration equivalent to a 2-week treatment period. The CHX containing mouth rinse was renewed after cycle 4, 7, 11, 14, 18, 21, and 25.

### Staining Solutions Preparation

The beverages were renewed after each cycle. The pH values of the beverages were determined using a pH meter (pH meter: Seven Multi, Mettler Toledo; pH electrode: InLab Routine Pro, Mettler Toledo, United States). Following beverages were used in this study: diet coke, diet lemonade, white wine, red wine, lager beer, black tea, coffee, black tea with milk, coffee with milk, ginger and lemon infusion, and water. Table 1 shows the preparation of the beverages and the pH value.

**Table 1:** Treatment groups

Beverage	Preparation	pH
Diet coke	Coke light (Coca-Cola company) was used as provided.	2.4
Diet lemonade	Diet lemonade (Schweppes) was used as provided.	2.9
White wine	Chardonnay (Gallo, California, 2016) was filtered to remove particles.	3.3
Red wine	Cabernet Sauvignon (high tannin content: Gallo, California, 2016) was filtered to remove particles.	3.4
Lager beer	Dark lager beer (Krombacher dark, Krombacher Brauerei Bernhard Schadeberg GmbH & Co. KG) was used as provided.	4.2
Black tea	A standard tea solution was prepared by adding one tea bag (Typhoo one cup, UK) per 50 mL of boiling water to a Duran bottle. After stirring at room temperature for 5 min using a magnetic stirrer, the tea bags were removed. After filtering through double layer gauze, the tea solution was stored at 50°C until use.	4.7
Coffee	A standard coffee solution was prepared by adding 1 g of instant coffee (Nescafe Classic) was mixed with 50 mL of boiled water for at least 15 min to completely dissolve the coffee crystals. After filtering through double layer gauze, the coffee solution was stored for use at 50°C.	4.7
Black tea with milk	5 mL of cow's milk (3.5% fat content, Weihenstephan GmbH & Co. KG, long life) was added to 100 mL of the tea solution.	4.8
Coffee with milk	5 mL of milk was added to 100 mL of the coffee solution.	4.8
Ginger and lemon infusion	A standard infusion was prepared by adding one tea bag (ginger lemon, Messmer) per 50 mL of boiling water in a Duran bottle. After stirring at room temperature for 5 min using a magnetic stirrer, the tea bags were removed. After filtering through double layer gauze, the tea solution was stored for distribution at 50°C.	5.8
Water	Volvic (Nestle) was used as provided.	7

### Experimental Approach

#### Staining Method without Brushing

The experiments were designed to simulate a 2-week (i.e., 14 days) treatment period and include a twice daily application of CHX-containing mouth rinse, resulting in a total of 28 staining cycles per testing. The experiments were performed over 2 days, that is, 14 staining cycles per day.

Samples ( *n* = 4) were stored in artificial saliva (2 minutes), rinsed with distilled water, exposed to the CHX mouthwash (according to the direction for use, 1 minute) and rinsed with distilled water. The samples were then placed in the beverages (staining media) for 10 minutes at 37°C and rinsed with distilled water. The specimens were air-dried after each cycle. Spectrophotometrical readings were taken after cycle 28 on dried samples ( Supplementary Fig. S1 , available in the online version).

#### Staining Method with Brushing

The above-mentioned model was then adapted to include a brushing step to mirror oral hygiene recommendation and understand how the different stains behave under mechanical stress. In these groups, an additional twice daily brushing step was simulated. The exposure time in each beverage was increased from 10 to 45minutes to allow a better differentiation between the beverages.

Cycle 1: Samples ( *n* = 4) were stored in artificial saliva (2 minutes), rinsed with distilled water, exposed to the CHX mouthwash (1 minute), and rinsed with distilled water. The samples were then placed in the beverage solution for 45 minutes at 37°C and rinsed with distilled water.
Cycles 2 to 28: The samples were then rinsed shortly in saliva and brushed with water or toothpaste for 4 seconds. Samples were stored in artificial saliva (2 minutes), rinsed with distilled water, exposed to the CHX mouthwash (1 minute), and rinsed with distilled water. The samples were then placed in the beverage solution for 45 minutes at 37°C and rinsed with distilled water. Specimens were air-dried after each cycle. Samples were air-dried overnight ( Supplementary Fig. S2 , available in the online version).

Toothpaste (Aquafresh triple protection fresh and minty, GlaxoSmithKline Consumer Healthcare GmbH & Co. KG) was mixed with distilled water to obtain 1:3 w/w slurries. In addition, only distilled water was used for brushing.

For brushing, the specimens were placed in the mechanical brushing device (toothbrush simulator ZM 3–8, SD Mechatronic, Germany). Before brushing, the toothbrushes were shortly wetted with water under standardized conditions. The toothbrushes (Oral B Indicator 35, medium, Procter & Gamble) were moved in reciprocating motions on the sample surface using the following conditions: brushing time 4 seconds (i.e., two cycles, whereas a cycle consists of a forward and backward stroke), and contact force of 1.5 N.

### Data Color Measurement

Images of the stained specimens were taken with a reflex camera (Canon EOS 600D, Japan) using standardized conditions.

Color measurement on an area of 5 mm diameter was done using a spectrophotometer (VITA Easyshade Compact, VITA Zahnfabrik, Germany). The color measurements were performed before and after staining to find the difference in stain and, where brushing took place, readings were taken after brushing. Values were recorded using the Hunter L*a*b* color scale, which is a color space with coordinates for lightness (white–black, L*), where the maximum for L* is 100 (a perfect reflecting diffuser) and the minimum would be 0 (black). The scale also represents redness–greenness (a*), where negative a* is green and positive a* is red, and yellowness–blueness (b*), where negative b* is blue and positive b* is yellow.

ΔE is the color difference between initial situation (before staining) and treated specimens (after staining or staining and brushing).



Statistical analyses of the ΔE mean values for different beverages were conducted by one-way analysis of variance with post hoc Tukey's honestly significant difference test and Levene's test for analyses of homogeneity of variance (Origin2019b, OriginLab Corporation Company, United States). The level of significance α was set to 0.05.

### Scanning Electron Microscopy Data

Scanning electron microscope (SEM) was used to characterize human enamel surfaces after staining and better understand the morphological changes of specimens. Therefore, the samples were coated with an ultra-thin carbon film by evaporation before the SEM analyses and analyzed with a SEM (Quanta 3D FEG from FEI, United States) equipped with an Everhart–Thornley detector capturing secondary electron images at 10 kV and at 5,000-fold magnification.

## Results

### Stain Formation without Brushing

**Fig. 1:**
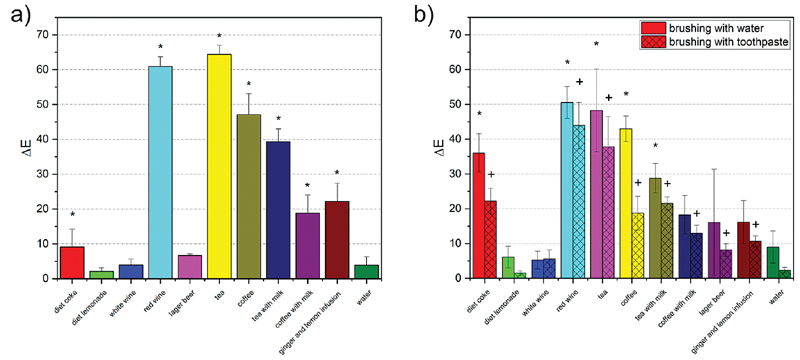
ΔE mean values (with standard deviation) of the human enamel samples after treatment with chlorhexidine in combination with different beverages, *in vitro* model: (a) without brushing; (b) with brushing; * and ^+^ , *p* < 0.05 when compared with water control.

Looking at the color measurements data ( Fig. 1A ), staining was low after treatment with diet lemonade, white wine, and lager beer compared with water ( *p* > 0.05). The staining potential of ginger and lemon infusion, coffee with milk, and diet coke was significantly higher compared with the aforementioned beverages and water ( *p* < 0.05). Highest staining was found for black tea, red wine, coffee, and tea with milk.

The reference values for the color measurements of the samples treated with CHX and water are in the expected order of magnitude for a negative control.

### Stain Formation with Brushing

A reduction of the stain for a given beverage could be observed between brushing with water and brushing with toothpaste ( Fig. 1B ). The model with brushing and without brushing cannot be directly compared as the exposure time in the beverage solutions differed.

After regular brushing with toothpaste and water was introduced, almost no staining was found after treatment with white wine, diet lemonade, and water control ( Fig. 1B ). High staining was found for black tea and red wine. An intermediate degree of staining was observed for tea with milk, coffee, coffee with milk, ginger and lemon infusion, lager beer, and diet coke. It was determined that there were no statistical differences between the water control and diet lemonade as well as white wine ( *p* > 0.05). It is noteworthy that brushing with toothpaste was more effective for coffee and diet coke than for tea and red wine.

### Scanning Electron Microscopy Data

**Fig. 2:**
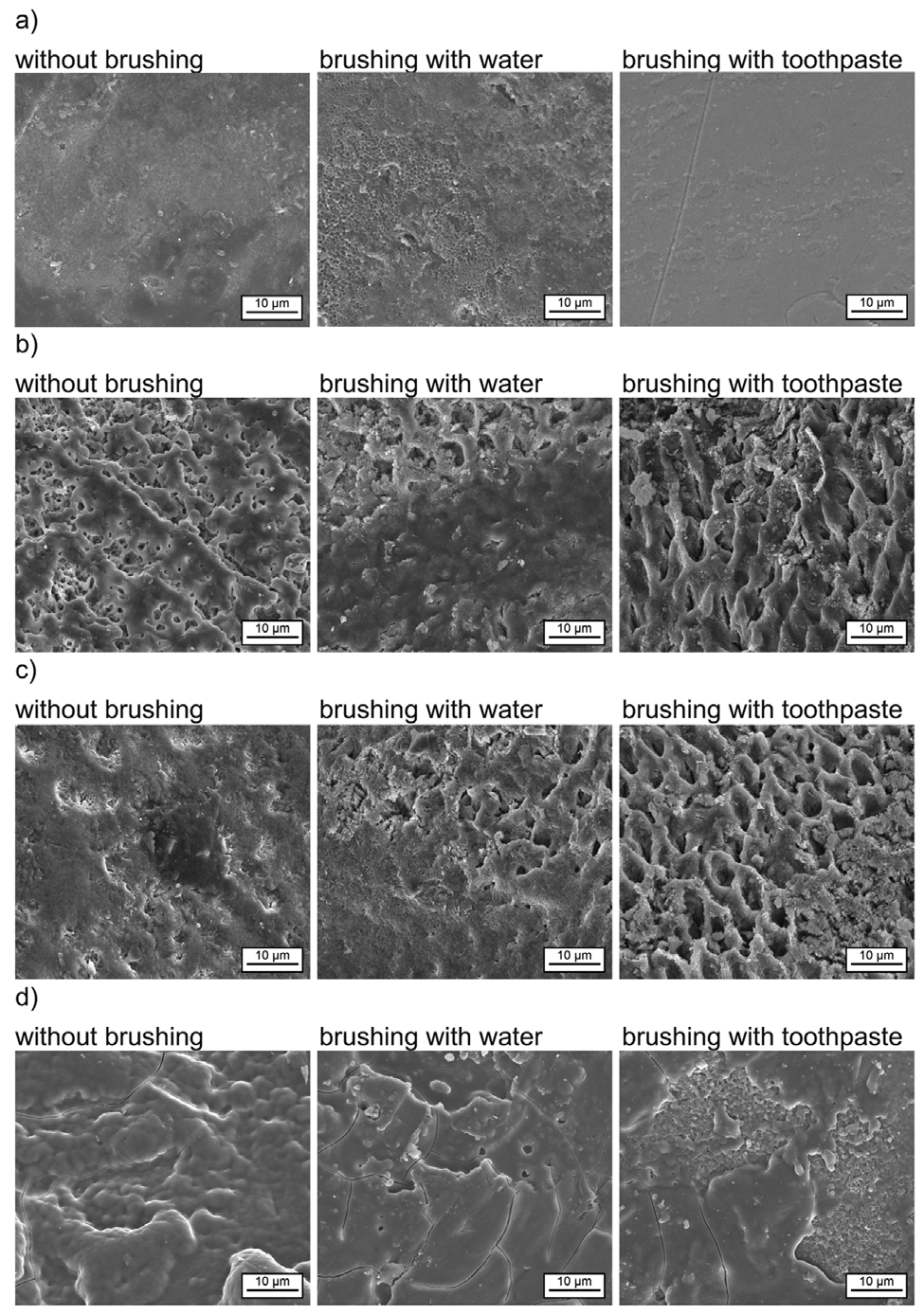
SEM images of the human enamel samples after treatment with chlorhexidine in combination with different beverages, *in vitro* model: (a) water (pH 7), (b) diet coke (pH 2.4), (c) white wine (pH 3.3), and (d) red wine (pH 3.4).

Figs. 2^3^ to 4 summarize the SEM obtained posttreatment with water, diet coke, white wine, red wine black tea, black tea with milk, coffee, and coffee with milk, respectively, after no brushing, brushing with water, and brushing with toothpaste.

**Fig. 3:**
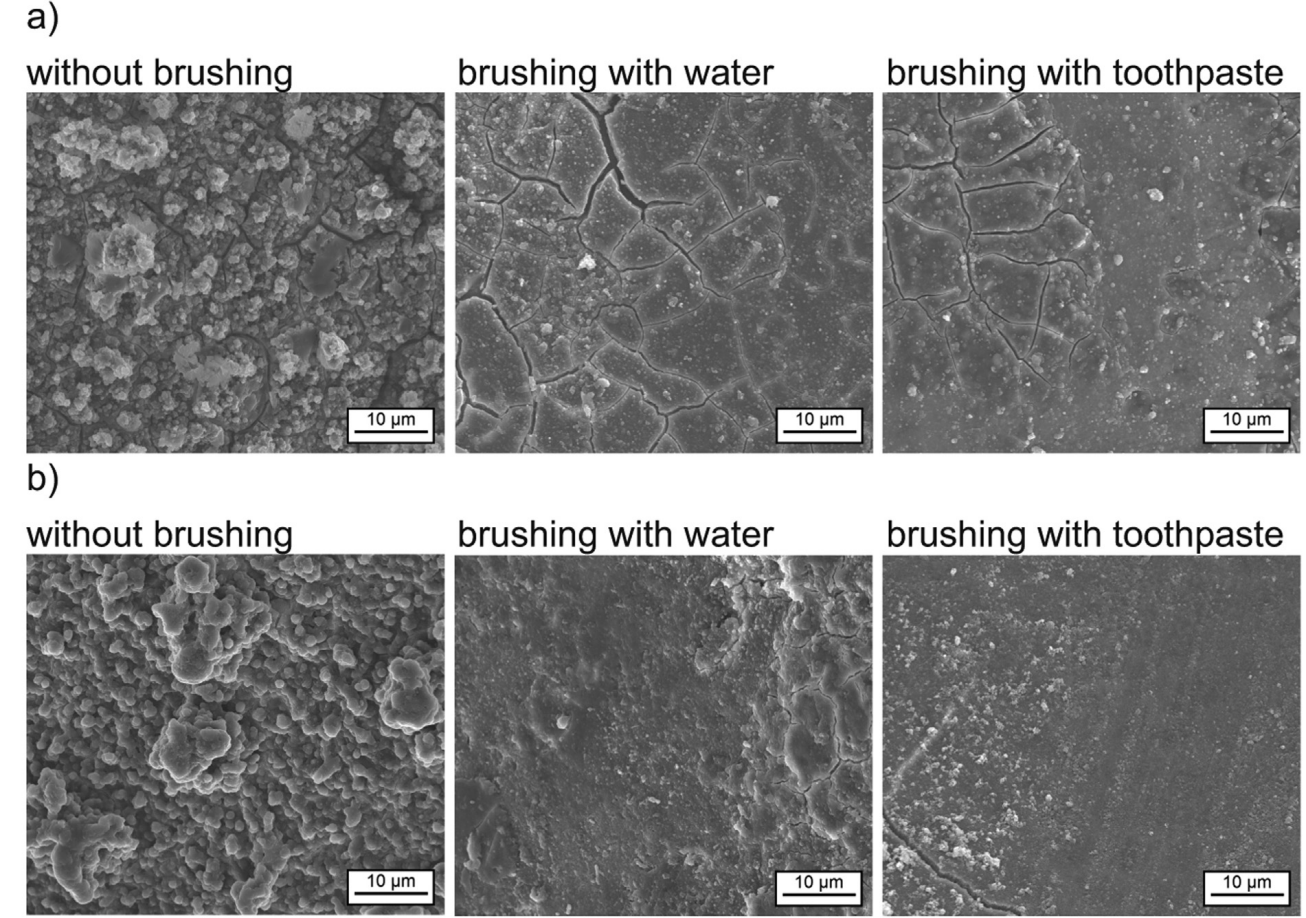
SEM images of the human enamel samples after treatment with chlorhexidine in combination with different beverages, *in vitro* model: (a) black tea (pH 4.7) and (b) black tea with milk (pH 4.8).

**Fig. 4:**
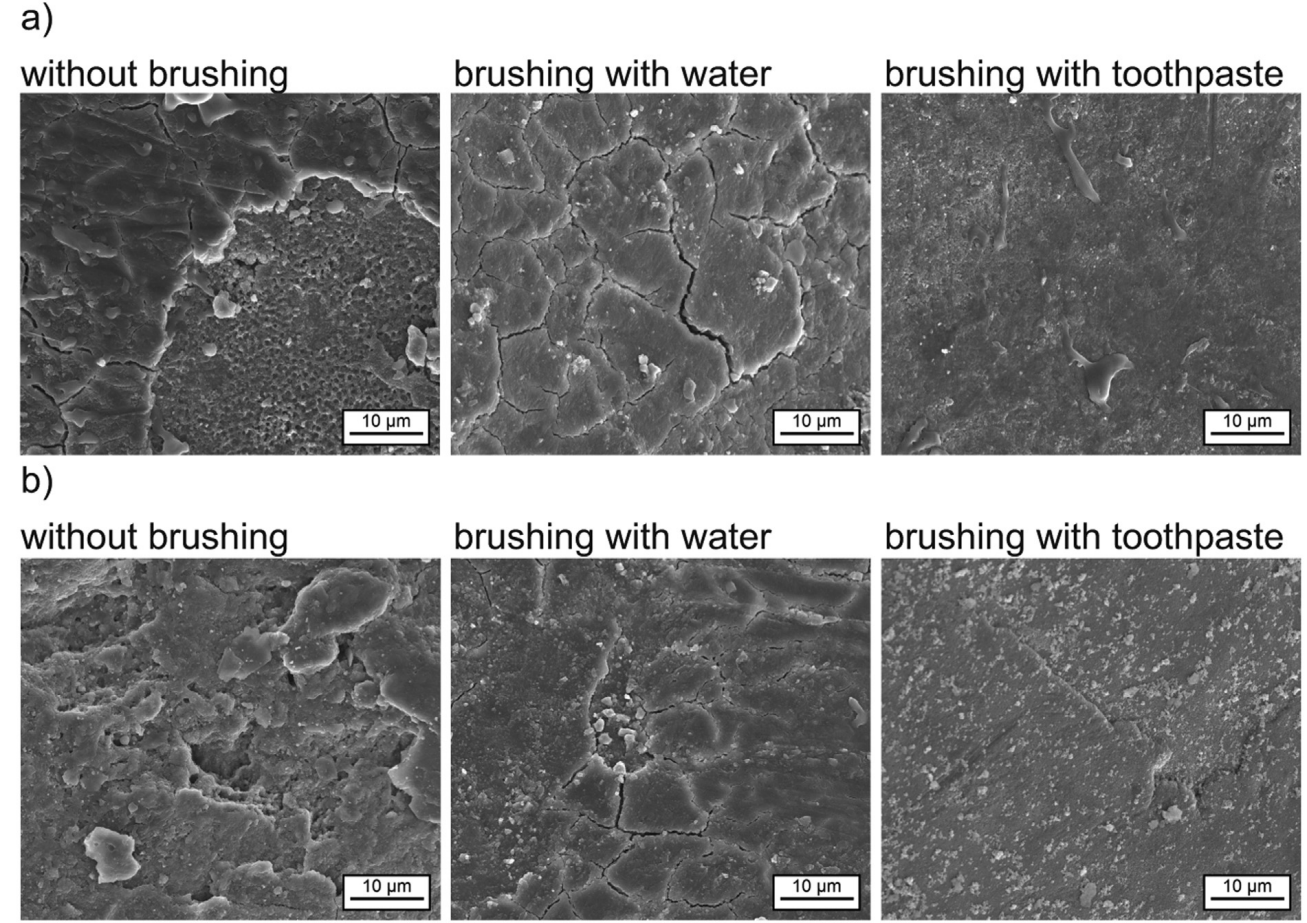
SEM images of the human enamel samples after treatment with chlorhexidine in combination with different beverages, *in vitro* model: (a) coffee and (b) coffee with milk.

The control sample treated with water only shows no surface layer as expected ( Fig. 2A ).

After brushing with toothpaste, the surface appears more smoothen which is probably caused by the abrading effect of the toothpaste.

Enamel samples treated with diet coke (pH 2.4) and white wine (pH 3.3) showed no surface layer, but had an extensively etched and roughened surface ( Fig. 2B, C ). After brushing with toothpaste, prism structures are clearly observable. This is probably caused by the mechanical impact of toothpaste which removes loosely adhering deposits and some porous demineralized material of the surface.

Enamel samples treated with red wine (pH 3.4) showed a continuous, rather smooth looking surface layer ( Fig. 2D ). The surface layer was still visible after brushing with water. Brushing with toothpaste causes some removal of this layer; however, the layer was still intact in some areas. The surface layer had a certain thickness as can be seen by the cracks (artifacts from sample preparation).

The SEM evaluations of enamel surfaces treated with black tea confirm the formation of a continuous surface layer ( Fig. 3A ). The surface layer was still visible after brushing with water and toothpaste. The surface layers have a certain thickness as can be seen by the cracks (artifacts from sample preparation).

Structural differences in the morphology of the surface layer formed by black tea in comparison to black tea with milk were clearly visible ( Fig. 3B ). The globular structures were much more pronounced after black tea treatment with milk. This difference in morphology of the surface layer could potentially be linked to the difference in staining potential. After brushing with toothpaste was introduced, the sample exhibited slight brushing traces. This implies that milk may play a role in the formation of the surface layer, making it less resistant to mechanical impact and explaining the observed lower staining potential of black tea with milk compared with black tea alone.

Enamel samples treated with coffee showed a surface layer ( Fig. 4A ). However, the surface layer did not cover the entire surface. As can be seen at the edges adjacent to noncovered enamel surface and by some cracks (artifacts from sample preparation) the stain has a certain thickness. The surface layer appeared to be resistant to brushing with water but not to brushing with toothpaste.

Enamel samples treated with coffee with milk show no continuous surface layer ( Fig. 4B ). The adherence of the stain was limited to some regions. This layer had a partly agglomerated structure and a certain thickness as could be seen by the cracks (artifacts from sample preparation). After brushing with toothpaste, the surface layer was completely removed except for some toothpaste residuals.

## Discussion

The present study is relatively complex due to the number of beverages tested and is partly exploratory in nature, as it investigated the staining potential of different beverages in combination with CHX, for which there are no data available in the current relevant literature. Most studies investigating the impact on dental discoloration caused by chromogens from food in combination with CHX have referred to tea or coffee. Addy et al have performed several studies to investigate and assess the staining potential of CHX in combination with tea in a cyclic treatment model. 3 7 10 11 12 In other studies, samples are stored continuously in the beverage of interest for several days or weeks. 13 14 From the authors' knowledge, data regarding the staining potential of lager beer, ginger and lemon infusion, etc. are not available.

Also, most of the staining studies were conducted with artificial materials (mostly polymethyl methacrylate) in contrast to the human dental crowns used here. 3 7 10 11 12 There are also only a limited number of *in vitro* studies available in which a brushing step was integrated to investigate the interplay between discoloration application and removal. 15 16

It is known from the literature that CHX alone does not stain but binds selectively to dietary chromogens, causing a colored layer. 17 The high staining intensity of black tea, coffee, and red wine in combination with CHX observed in this study agrees with previous studies, and several theories have been stated in the literature to explain this stain formation. For example, CHX produced an increase in tea staining in a study on recently extracted human teeth and supported the hypothesis that CHX staining is due to a precipitation reaction between dietary chromogens and adsorbed CHX. 18 Furthermore, another study has revealed that tea and CHX alone bind in small quantities to hydroxyapatite, but when added in combination, binding of both to hydroxyapatite was highly increased. 19 It was observed that the acquired pellicle over the tooth reduced CHX and tea binding, but conversely increased the binding of either tea or CHX alone to hydroxyapatite. 19 CHX which was adsorbed to the acquired pellicle caused modification of the pellicle properties, leading to a subsequent increase in adsorption of salivary and black tea components which ultimately led to an increased staining of the pellicle. 20 The present results show that the samples treated with coffee produced less staining than black tea. This effect has also been described in the literature, the least staining coffee and least staining tea brands were approximately triplicate less chromogenic than the most staining equivalent beverage. 21 Surface morphology evaluation by SEM suggests that surface layers caused by coffee are more easily removed by brushing than those caused by black tea and red wine. SEM also confirmed that the addition of milk to black tea and (probably) coffee changes the morphology of the surface layer making it less resistant to brushing which agrees with the literature.

The samples treated with black tea with milk exhibited slight brushing traces, in comparison with the black tea only samples. The black tea stain is more cohesive and more resistant to mechanical stress (i.e., brushing). This could mean that some milk components play a role in the formation of the surface layer making it less resistant to mechanical impact. Such an effect could explain the observed lower staining potential of black tea with milk compared with black tea alone. A published study investigated the component of milk that is responsible for milk's stain reducing properties and concluded that the addition of milk to tea significantly reduces the tea's ability to stain teeth. Casein was determined to be the component of milk that is responsible for preventing tea-induced staining of teeth. 14

Several previous published studies showed that highly pigmented beverages with a low pH value cause extrinsic tooth discoloration. 20 22 A low pH value from acidic drinks has been found to increase the roughness of the tooth enamel surface rendering enamel more prone to staining and facilitate easier deposition of chromogenic agents from pigmented beverages. 23 It was further supported by a study in which acidic food colorant solution caused significant tooth discoloration compared with highly pigmented neutral or alkaline solutions suggesting that the degree and type of tooth discoloration are found to be influenced by both the low pH and food color rather than the dietary pigment alone. 22

It is also known from literature that carbonated drinks such as coke demonstrated strong brown colored discoloration in the enamel of the tooth. 13 Staining of teeth is mainly due to two substances namely phosphoric acid and chromogens. Phosphoric acid weakens the tooth enamel and the chromogens act on the etched enamel and in turn, cause a brownish discoloration.

Red wine stained the teeth more than white wine in an *in vitro* study. 24 Higher tannin content in red wine has been found to increase the intensity of stain formation. It remains unclear whether tannic acid alone or if also other phenolic components cause discoloration and how they facilitate it. 22 In spite of the low pH value, SEM images of red wine–treated samples showed no hints for surface erosion. The authors assume that the effects caused by acidity of red wine are not visible due to the stain layer formation. Wine has been found to produce extrinsic dental staining by leaving the surface of the teeth more susceptible to the action of the extrinsic chromogens. The staining potential of diet coke was comparably high on human enamel, particularly in test series with brushing. The high staining potential on enamel is probably connected to its low pH-value. The enamel surfaces seem to be slightly etched due to the treatment.

## Conclusion

The *in vitro* staining protocol used in this study, which was adapted to work with and without a brushing step, is appropriate to evaluate dental discolorations caused by different beverages. The beverages could be placed in groups according to their staining potential; the highest staining potential was confirmed for black tea and red wine which agrees with the literature.

Slight to medium discolorations were found after treatment with ginger and lemon infusion, coffee, coffee with milk, tea with milk, and lager beer. The staining potential of white wine and diet lemonade was very low and close to the water control.

This study showed that brushing with toothpaste is more effective in removing stain but does not change the order of staining potential of given staining dietary elements. The data help understand the complexity of CHX's staining mode of action, its contribution to stain overtime, and the potential to improve recommendation protocols. The visual data may help demonstrate that appropriate patient's guidance can avoid stain and in turn help improve patient compliance during short-term use of this gold standard antimicrobial treatment.
