# Supplementary material for: The Impact on Dental Staining Caused by Beverages in Combination with Chlorhexidine Digluconate
Source: Eur J Dent. 2022 Feb 23;16(4):911–8. doi: 10.1055/s-0041-1742123 (PMC9683888; doi:10.1055/s-0041-1742123)
Supplement: Supplementary file 1 — Supplementary Material [file 10-1055-s-0041-1742123-s2191701.pdf]

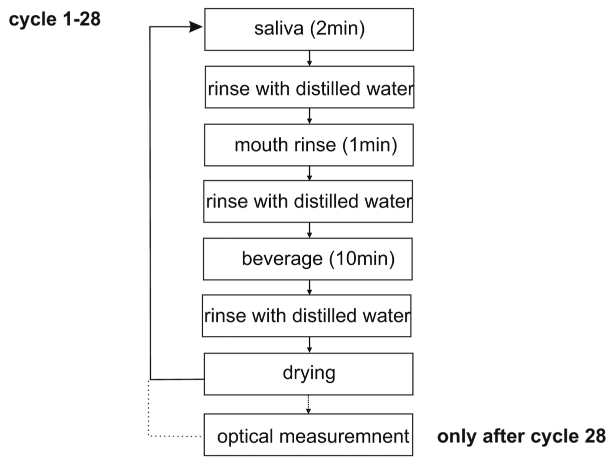

Supplementary Fig. S1 Testing procedure without brushing.

 © 2022. The Author(s).

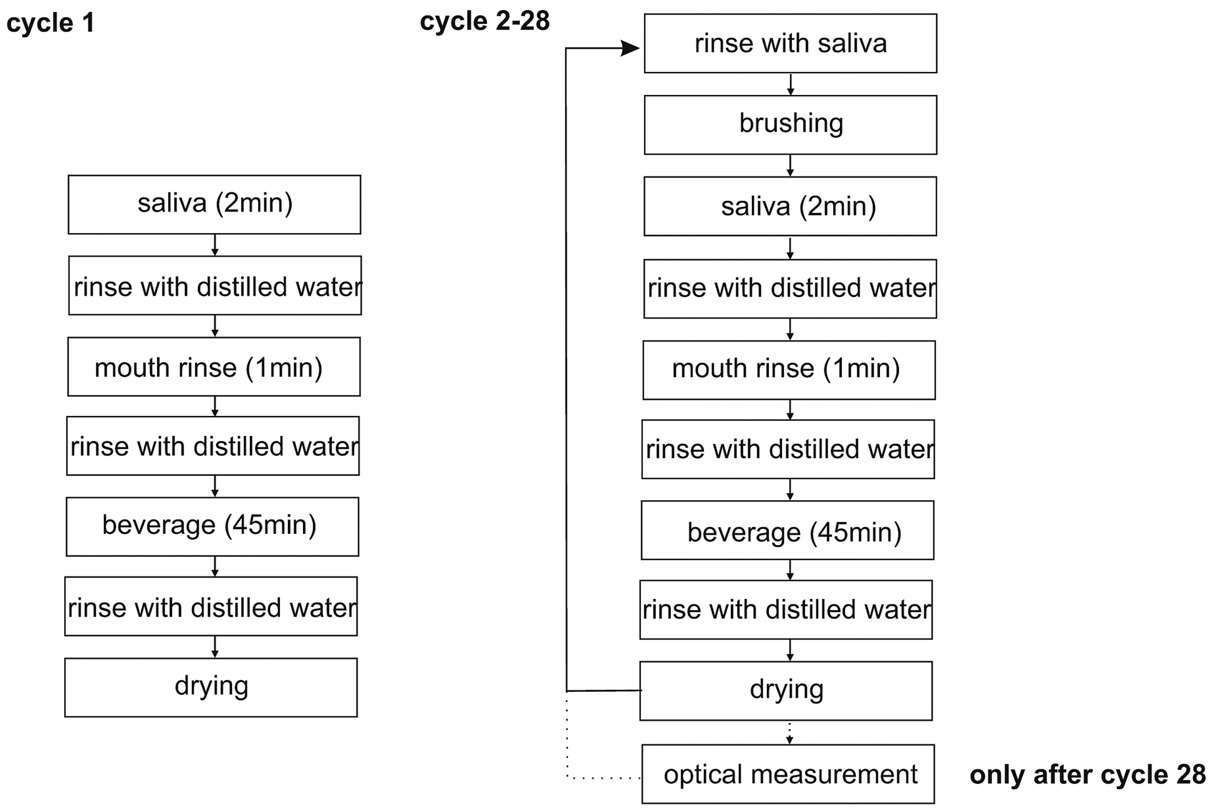

Supplementary Fig. S2 Testing procedure with brushing.
